# Sodium-Ion Battery: Can It Compete with Li-Ion?

**DOI:** 10.1021/acsmaterialsau.3c00049

**Published:** 2023-07-27

**Authors:** Haegyeom Kim

**Affiliations:** Materials Sciences Division, Lawrence Berkeley National Laboratory, Berkeley 94720, California, United States

**Keywords:** Batteries, Sodium, Cathodes, Energy Storage, Lithium, Critical Element

## Abstract

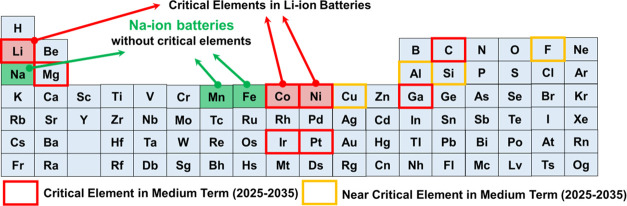

As concerns about the availability of mineral resources for lithium-ion batteries (LIBs) arise and demands for large-scale energy storage systems rapidly increase, non-LIB technologies have been extensively explored as low-cost alternatives. Among the various candidates, sodium-ion batteries (SIBs) have been the most widely studied, as they avoid the use of expensive and less abundant elements such as lithium, cobalt, and nickel while also sharing similar operating principles with LIBs. In this Perspective, we discuss why SIBs hold great promise and can act as competitors to lithium-ion technology. In addition, the remaining challenges and future research directions are highlighted, focusing on cathode developments and the use of SIBs in large-scale applications, including electric vehicles and stationary energy storage.

## Low-Cost Alternative Sodium-Ion Batteries

Li-ion battery (LIB) technology currently powers electric vehicles (EVs), helping to make an important transition to a sustainable energy society. According to the U.S. Energy Information Administration (EIA), the transportation sector produces 37% of the CO_2_ emissions in the United States, which is the highest among the four end-user sectors (transportation, industrial, residential, and commercial).^1^ Therefore, electrification of the transportation sector is critical to achieve the goal of “net-zero emissions” by 2050.^2^ Significant progress has been made in LIB technology in recent decades, with both the specific energy (Wh/kg) and energy density (Wh/L) of LIBs significantly increasing and the LIB pack cost dramatically decreasing (from $732/kWh in 2013 to $150/kWh in 2022).^3^ Driven by these technical advancements as well as government policy, global EV sales have increased rapidly from a few thousand in 2010 to over 6 million in 2021, with this number projected to reach over 45 million by 2030.^4^ In addition, the demand for stationary energy storage systems continues to increase. It is thus inevitable to question whether LIB technology alone can meet such increasing demands for large-scale energy storage systems.

**Figure 1:**
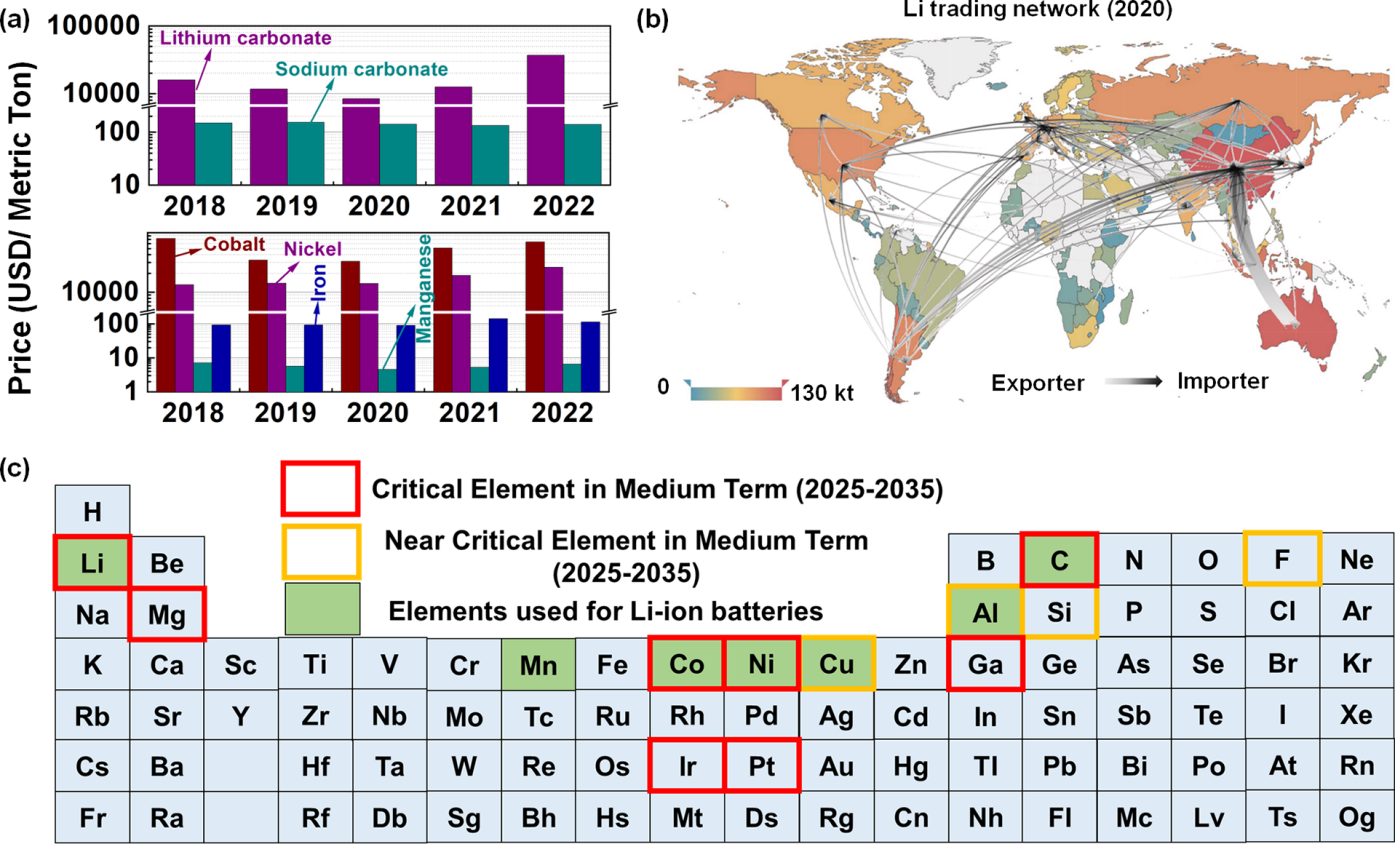
Price of elements used in lithium-ion and sodium-ion cathode materials. (a, Top) Price (U.S. dollars per metric ton) of lithium and sodium resources. U.S. Geological Survey (USGS) Minerals Information: Commodity Statistics and Information (accessed May 2023). (a, Bottom) Price (U.S. dollars per metric ton) of cobalt, nickel, manganese, and iron resources. U.S. Geological Survey (USGS) Minerals Information: Commodity Statistics and Information (accessed May 2023). (b) Trade network of lithium in 2020. The colors of countries represent the total trade volume of a country. The width of the edge represents the volume of trade, and the thicker edges indicate a larger volume of trade between the two countries. Adapted with permission from ref (6). Copyright 2023 Elsevier. (c) Critical elements and near critical elements in medium term (2025–2035), which are defined by the U.S. Department of Energy (data accessed July 2023). Graphite is defined as a critical element among various forms of carbon. Elements that are widely used in LIBs are also highlighted.

There has been a significant rise in the price of lithium carbonate in recent years (top of Figure 1a) as well as in that of cobalt (Co) and nickel (Ni) resources (bottom of Figure 1a) since 2020.^5^ This trend will be even further accelerated, which can be a huge obstacle to moving forward to a green society. In addition, lithium resources are not uniformly distributed in the world, and the lithium mining and LIB manufacturing sites are not always in the same country. Therefore, additional cost needs to be paid for lithium trade (Figure 1b).^6^ Very recently, the U.S. Department of Energy published lists of critical and near critical elements in medium term (2025–2035) given their importance to energy and supply risk as shown in Figure 1c.^7^ Essential elements of LIBs, including Li, Co, and Ni, are included in the lists. In this regard, SIBs have recently attracted much attention as alternative cost-effective energy storage systems.^8−10^ Sodium carbonate is much more abundant and cheaper than its Li counterpart (top of Figure 1a).^5^ In addition, we have more diverse options for cathode selection for SIBs. The layered oxide cathodes for LIBs necessitate the use of expensive cobalt and nickel to maintain the ordered layered structure, where the lithium and transition metals are separated in distinct layers. In contrast, the formation of sodium-based layered transition-metal oxides is thermodynamically favorable with various transition metals, including low-cost manganese (Mn) and iron (Fe), because the sodium and transition metals tend to be separated because of their large ionic size difference. Therefore, Mn and Fe could be used in cathode materials for SIBs, significantly reducing the material cost.^11^ In addition, in SIBs, nonlayered compounds, such as polyanionic compounds and Prussian blue analogues also show comparable electrochemical performance without the use of expensive Co and Ni.^12−15^

## Can SIBs Be a Competitor to LIBs?

**Figure 2:**
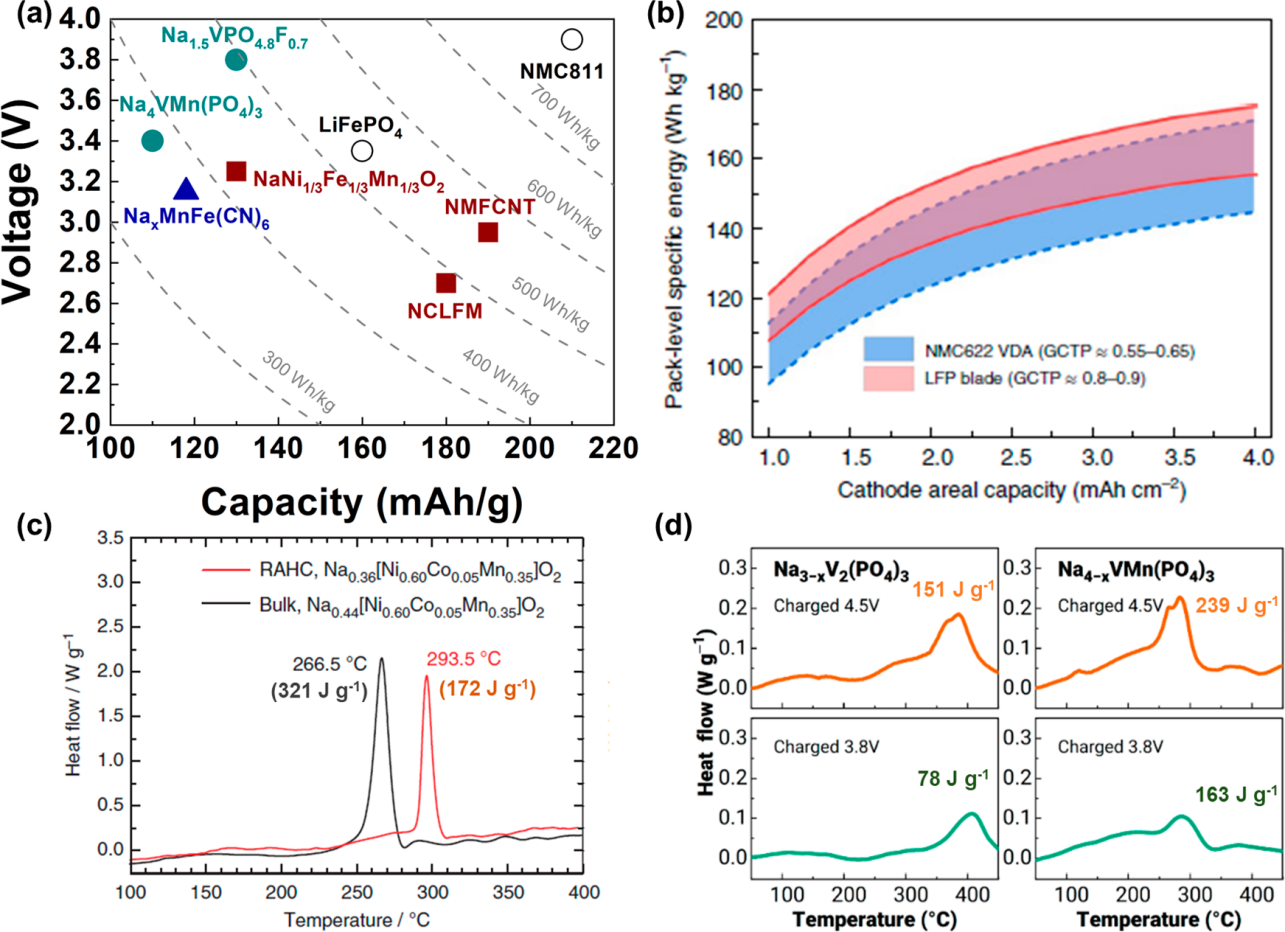
(a) Specific capacity (mAh/g)–voltage (V) plots of the LIB and SIB cathodes. The data were obtained from discharge profiles in the literature.^11,26−32^ NMFCNT: NaMn_0.2_Fe_0.2_Co_0.2_Ni_0.2_Ti_0.2_O_2_ and NCLFM: Na_0.75_Ca_0.05_Li_0.15_Fe_0.2_Mn_0.6_O_2_. The voltage for Li cathodes is plotted vs Li metal, and that for Na cathodes is plotted vs Na metal. (b) Evolution of the gravimetric specific energy of the LiFePO_4_ blade battery and NMC622 prismatic battery at the pack level as a function of the cathode areal capacity. GCTP stands for gravimetric cell-to-pack ratio. Adapted with permission from ref (20). Copyright 2021 Springer Nature. (c) Differential scanning calorimetry (DSC) results of the sodium transition-metal oxide cathode at charged states. Adapted with permission from ref (24). Copyright 2015 Springer Nature. (d) DSC curves for the charged electrodes containing Na_3_V_2_(PO_4_)_3_ and Na_4_VMn(PO_4_)_3_ cathode materials at different potential cutoffs (3.8 V on the bottom and 4.5 V on the top). Reprinted with permission under a Creative Commons CC-CY License from ref (25). Copyright 2023 MDPI.

It is widely accepted that SIBs are a cost-effective option for energy storage, in particular, stationary energy storage systems. However, it remains debatable whether the specific energy (Wh/kg) and energy density (Wh/L) of SIBs are sufficient for EV applications. At the electrode level, these values are lower than those of state-of-the-art LIB cathodes (*i.e*. NMC811 or LiNi_0.8_Mn_0.1_Co_0.1_O_2_), as illustrated in Figure 2a. Yet, notably, many cathode materials for SIBs exhibit specific energy (Wh/kg) and energy density (Wh/L) comparable to those of LiFePO_4_, which has recently been considered a strong competitor of NMC cathode materials. Several EV manufacturers have adopted or plan to adopt LiFePO_4_ as a cathode material.^16,17^ One main reason that the LiFePO_4_ cathode is posed to be a strong competitor for NMC cathodes in the EV market is its high safety. Whereas NMC811 has a low onset temperature (∼230 °C) for exothermic reactions and exhibits a high heat release (>900 J/g),^18^ LiFePO_4_ has a much wider but flatter exothermic reaction peak at 250–350 °C and exhibits a significantly lower heat release (∼150 J/g).^19^ In LiFePO_4_, the strong P–O covalent bond inhibits oxygen release compared with that in layered oxide NMC cathodes, thereby improving the thermal stability. This improved thermal stability of LiFePO_4_ enables an increase in the cell-to-pack ratio of the batteries, which will increase the pack-level specific energy (Wh/kg) and energy density (Wh/L). In addition, the high thermal stability of LiFePO_4_ allows elevated temperatures to operate, which also increases the pack-level specific energy (Wh/kg) and energy density (Wh/L). Therefore, LiFePO_4_-based battery pack can exhibit similar to or even higher specific energy (Wh/kg) than that of a battery pack using NMC cathodes (Figure 2b).^20^ Similarly, high specific energy (Wh/kg) and energy density (Wh/L) of SIBs could be achieved at the pack level when using cathode materials with high thermal stability. It is notable that some SIB companies already reported significantly improved cell-level specific energy comparable to LiFePO_4_-based LIBs.^21^ Looking at the success of LiFePO_4_, it is also expected that SIB will hold better promises in applications where cost is more important than the specific energy or energy density such as stationary energy storage systems.

Unlike in the Li-ion system, the layered oxide compounds and polyanionic compounds in the Na-ion system exhibit comparable specific energies (Wh/kg), as illustrated in Figure 2a. The sodium-based layered oxide cathodes possess a sloped voltage profile, which limits the specific capacity and lowers the average voltage. This intrinsic limitation is well documented in our previous publications.^22,23^ Although Prussian blue analogues deliver comparable specific energy (Wh/kg), their low material density (∼1.8 g/cm^3^) prohibits their use in applications where the energy density (Wh/L) is critical such as in EVs. Because polyanionic compounds exhibit high specific energy (Wh/kg) and energy density (Wh/L), similar to those of sodium-based layered oxides, we expect that the polyanionic compounds could be a better choice for use in EVs given their higher thermal stability. In particular, phosphates and their derivatives possess greatly improved thermal stability due to the strong P–O covalent bond similar to that in LiFePO_4_. For example, sodium transition-metal oxide exhibits a very sharp exothermic peak at 250–300 °C at a charged state (Figure 2c).^24^ The amount of heat release is over 300 J/g without engineering. Importantly, Na_3_V_2_(PO_4_)_3_ shows a flatter exothermic peak at ∼400 °C, with much smaller heat release of 78 J/g when charged up to 3.8 V, as demonstrated in Figure 2d.^25^ At the over charged state (up to 4.5 V), the heat release increases to 151 J/g. Na_4_VMn(PO_4_)_3_ exhibits a higher heat release of 163 J/g than Na_3_V_2_(PO_4_)_3_ when charged to 3.8 V (within the normal operation cutoff); however, this value is still lower than that of layered oxide cathodes. Therefore, we anticipate that SIBs using polyanionic compound cathodes can use the battery pack design with a high cell-to-pack ratio similar to the LiFePO_4_ system, which will deliver higher pack-level specific energy (Wh/kg) and energy density (Wh/L) than those based on layered oxides. In addition, we will be able to increase the operating temperature of SIB to improve the specific energy (Wh/kg) and energy density (Wh/L) when polyanionic compounds cathodes with high thermal stability are used. We expect that SIBs relying on polyanionic cathode materials with high thermal stability will show great promise for large-scale systems, in particular for midrange EVs and stationary energy storage.

## Outlook

**Figure 3:**
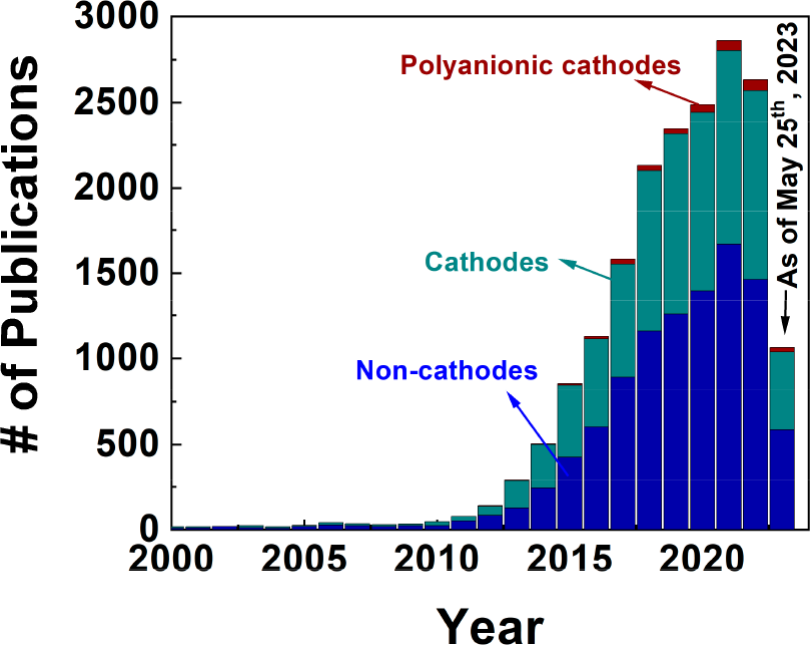
Number of publications on SIBs over time. Data obtained from Web of Science. Accessed on May 25, 2023.

Although polyanionic compounds have shown great promise in SIBs because of their high specific energy, energy density, and thermal stability, only a small portion of studies have explored sodium-based polyanionic cathode materials (Figure 3). Furthermore, most of those studies focused on vanadium(V)-based compounds such as Na_3_V_2_(PO_4_)_3_ and Na_3_V_2_(PO_4_)_2_F_3_ and their derivatives. However, vanadium-based cathode materials are not the best option for low-cost rechargeable batteries because of the relatively high cost and low abundance of vanadium resources. More efforts should thus be devoted to developing cost-effective Mn- and Fe-rich polyanionic cathode materials for SIBs,^12−14,33^ which will make the SIB system more competitive. Reported Mn- and Fe-rich polyanionic cathode materials for SIBs include but not limited to Na_4_MnCr(PO_4_)_3_,^33^ NaFeSO_4_F,^12^ Na_4_Fe_3_(PO_4_)_2_(P_2_O_7_),^13^ and Na_2+2x_Fe_2–x_(SO_4_)_3_.^14^

Because thermal stability is an essential enabler to make polyanionic cathode-based SIBs competitive with LIBs, as discussed in the previous section, researchers should investigate the thermal stability and thermal runaway properties more carefully. These efforts should not be limited to the electrode level but need to be expanded to the full-cell level, including the electrolyte and anode, and even to the pack level.

Prussian-blue-analogue-based SIBs could be used for stationary energy storage systems because of their use of low-cost Mn and Fe redox elements and because the stationary system is relatively less sensitive to the energy density (volumetric energy) compared to EVs. However, the safety of Prussian blue analogues is still barely understood. It could be argued that they do not have oxygen to be released and that no thermal runaway is expected. However, these materials contain large amounts of cyanide (CN) ligands in the structure, and it remains unclear how those cyanide ligands will react at elevated temperatures, in particular, at charged states with flammable organic electrolytes. In fact, the decomposition products of Prussian blue cathodes include hydrogen cyanide (HCN) and cyanogen ((CN)_2_), which are extremely flammable. When thermal runaway starts, these decomposition products will be extremely reactive and can lead explosion. Therefore, more in-depth studies to understand the thermal stability and thermal runaway properties of Prussian blue analogue-based SIBs are thus required.
